# Unexpected Twists: Electrophysiological Correlates of Encoding and Retrieval of Events Eliciting Prediction Error

**DOI:** 10.1111/psyp.14752

**Published:** 2024-12-30

**Authors:** Gözem Turan, Veronika Spiertz, Oded Bein, Yee Lee Shing, Sophie Nolden

**Affiliations:** ^1^ Department of Psychology Goethe University Frankfurt Frankfurt Am Main Germany; ^2^ IDeA Center for Individual Development and Adaptive Education DIPF Leibniz Institute for Research and Information in Education Frankfurt am Main Germany; ^3^ Princeton Neuroscience Institute Princeton University Princeton New Jersey USA; ^4^ Department of Psychiatry, Weill Cornell Medicine Weill Cornell Institute of Geriatric Psychiatry New York New York USA

**Keywords:** electroencephalography, episodic memory, event‐related potentials, prediction error, predictive processing, statistical learning

## Abstract

According to the predictive processing framework, our brain constantly generates predictions based on past experiences and compares these predictions with incoming sensory information. When an event contradicts these predictions, it results in a prediction error (PE), which has been shown to enhance subsequent memory. However, the neural mechanisms underlying the influence of PEs on subsequent memory remain unclear. This study investigated the electrophysiological correlates during encoding and retrieval of events eliciting PEs. We employed a statistical learning task in which participants were presented with pairs of objects in sequence. Subsequently, while recording electroencephalography (EEG), we introduced PEs by replacing the second object of each pair with new objects and we then tested the participants' memory. Behaviorally, PEs did not enhance memory. During retrieval, we observed higher amplitudes in the recollection‐related late positive component for violation items that were remembered compared to those that were forgotten. In contrast, no evidence for the presence of the FN400 component associated with familiarity was found. These results suggest that recollection, but not familiarity, plays a crucial role in the interplay between PE and memory. Contrary to our hypothesis, we did not observe a relationship between PEs and the P3 component during encoding. In conclusion, our study contributes to the growing body of knowledge concerning the intricate relationship between PEs and episodic memory. It sheds light on the underlying neural mechanisms involved and emphasizes the importance of recollection in this context.

## Impact Statement

Our study investigates the neural mechanisms underlying the effects of prediction errors (PE) on memory processes, specifically encoding and retrieval. In a statistical learning paradigm, predictions were violated, and their memory was tested. Contrary to our expectations, behavioral results did not demonstrate a memory advantage for PE‐induced events. Nonetheless, our findings highlight the significance of recollection in the link between PE and memory.

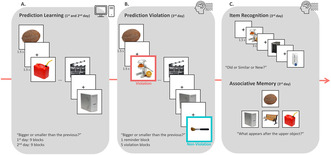

## 1 Introduction

Although the first season of Game of Thrones was broadcast 12 years ago, many viewers still remember the execution of the main character, Ned Stark. According to storytelling conventions, the viewers of the show might have predicted that the protagonist would ultimately be spared or that justice would be served in the end. However, when Ned was beheaded in a sudden twist, it violated the viewers' prediction. This violation might have led the viewers to process the unexpected event in a distinctive way since it differed from their prediction. Distinctive processing could explain why Ned's execution remains such a memorable event, illustrating the role of prediction error (PE) in memory processes. Indeed, whether and how PEs modulate memory is a topic of intense investigation in cognitive psychology and neuroscience (Aitchison and Lengyel 2017; Bein et al. 2023; Ergo, De Loof, and Verguts 2020; Quent, Henson, and Greve 2021). Here, we investigated the electrophysiological correlates of encoding and retrieval of PE to gain a better understanding of the relationship between PE and memory.

According to the predictive processing framework, our brain constantly predicts likely occurrences based on past experiences (Bar 2007; Friston 2010; Henson and Gagnepain 2010). The brain continually compares sensory information with its predictions. When a prediction is confirmed, it reinforces existing internal models and increases confidence in future predictions. Conversely, when a prediction is violated, a PE occurs, signaling the need for additional processing to update predictions. This way, the brain utilizes PEs to adaptively refine its predictions over time. However, we have limited knowledge regarding how the brain processes events that give rise to PE and how the underlying mechanisms contribute to subsequent memory.

Preceding studies have demonstrated that unexpected events typically improve memory (e.g., von Restorff (1933) effect for isolated events). Recent research is in line with this tradition by showing that PEs facilitate memory. These studies have suggested that events accompanied by PEs contain significant information that requires enhanced encoding for memory (Bein, Plotkin, and Davachi 2021; Bein et al. 2023; Brod, Hasselhorn, and Bunge 2018; Kafkas and Montaldi 2018; Quent, Greve, and Henson 2022). Improved encoding of events that elicit PEs might generate detailed ‘snapshots’ of these events, resulting in a memory advantage (Henson and Gagnepain 2010). Additionally, PEs might enhance pattern separation, a process by which distinct memory traces are created, potentially separate from those associated with previous predictions (Frank, Montemurro, and Montaldi 2020). It has been concluded that PEs enhance memory by rendering events that elicit PEs more distinctive, and this effect was supported by representational similarity analysis on neural network analyses of the hippocampus (Aisa, Mingus, and O'Reilly 2008; Frank, Montemurro, and Montaldi 2020), indicating enhanced pattern separation in various hippocampal subregions. Furthermore, according to event segmentation theory (Zacks et al. 2007), which addresses how continuous experience is separated into discrete events, PEs trigger an upregulation of attentional resources toward the specific event. This increased attention enables the brain to process information more deeply and prompts the identification of an event boundary, potentially leading to the separation of events and robust memory. Triggering an event boundary in this manner aids in segmenting the continuous stream of sensory information into discrete events and facilitates subsequent memory benefits (Wahlheim et al. 2022). Notably, as attention increases, neural similarities within events, compared to the across event boundaries, tend to grow, emphasizing the role of pattern completion within events, and this underscores the hippocampus's role in supporting predictions during the unfolding of events (Bein and Davachi 2022; Paz et al. 2010; Schapiro, Kustner, and Turk‐Browne 2012). Yet, it should be noted that while recognizing the role of event segmentation in memory for preceding events, we focus on memory of events that elicit PE. To summarize, previous research suggests that events giving rise to PE are encoded more effectively and result in better memory.

In addition to encoding, retrieval processes might also contribute to how PE enhances memory. For example, a study by Kafkas and Montaldi (2018) investigated the effects of PE during encoding and retrieval. Their results revealed that predicted events enhanced familiarity, which refers to a subjective feeling that an event has been experienced before, while unpredicted events enhanced recollection, which involves the retrieval of specific episodic details (Cowell, Barense, and Sadil 2019). This finding aligns with the framework proposed by Henson and Gagnepain (2010), which suggests that predictive events aided by familiarity benefited during the retrieval. Conversely, unpredicted events elicit a memory characterized by snapshot‐like details, leading to enhanced recollection. The connection between encoding and retrieval processes and the impact of PEs is further supported by the concept of selective retrieval process, namely, intentionally recalling specific information while excluding other related or unrelated information (Lu, Hasson, and Norman 2022). This theory suggests that error signals, even during encoding, may play a vital role in facilitating subsequent recollection during retrieval (Fenerci and Sheldon 2022; Wahlheim et al. 2022). Moreover, previous research suggests that the occurrence of events deviating from previous knowledge can trigger engagement in brain regions associated with successful retrieval, such as cortical and hippocampal memory networks (for a review, Alonso et al. 2020). Taken together, these findings suggest that the effects of PEs on memory are not limited to the encoding stage but can also extend to the retrieval phase.

Notwithstanding the importance of the aforementioned studies, PEs might not always enhance memory. A recent body of research has consistently reported that PEs do not guarantee subsequent memory advantage (Ortiz‐Tudela et al. 2023; Turan et al. 2023). For instance, in one study, participants were asked to make explicit predictions regarding associations between sequentially presented pairs, and these predictions were either met or violated in varying levels of PEs. The results revealed better recognition memory for items that were consistent with participants' predictions but not for items eliciting PEs. These results are consistent with prior work showing better memory for expected compared to unexpected events, indicating a memory congruency effect (Alba and Hasher 1983; Brod and Shing 2019; Craik and Tulving 1975; Liu, Grady, and Moscovitch 2018; Ortiz‐Tudela et al. 2017). Thus, the effect of PEs on subsequent memory is not straightforward and further exploration is warranted. Currently, there is limited empirical evidence regarding the reliable conditions under which PEs facilitate memory, highlighting the need for additional research.

Through the investigation of how the brain processes PEs and how its underlying operations influence subsequent memory, we can enhance our understanding of the effects of PEs and potentially reconcile the divergent findings in the literature. Event‐related potentials (ERPs) can provide an ongoing evaluation of neural processes that correlate with PEs. By comparing the time‐locked changes in the brain's electrophysiological activity in response to violating events that are later remembered versus later forgotten, we can identify neural processes that contribute to subsequent memory enhancement for PEs. For instance, the P3 component has been one of the highly studied ERP components which was traditionally associated with oddball signals (Polich 2007), attention (Kramer, Wickens, and Donchin 1985), evaluation of novelty (Friedman, Cycowicz, and Gaeta 2001), and context updating (Donchin 1981). It has also been demonstrated that P3 amplitude is an indicator of successful subsequent memory (Fabiani, Karis, and Donchin 1986; cf. Höltje and Mecklinger 2022; Rangel‐Gomez and Meeter 2013). This implies that memory‐related changes in P3 amplitude might index encoding processes associated with PEs that facilitate subsequent memory. Furthermore, in addition to the mentioned traditional origins, P3 has also been linked to reward PEs (see a recent meta‐analysis, Stewardson and Sambrook 2020), novelty processing influenced by expectations (Schomaker and Meeter 2018) and hierarchical violations as suggested by the predictive coding theory (Vidal‐Gran et al. 2020). Even though these studies demonstrated the associations between P3 and the processing of violation (i.e., PEs), it is still unclear whether P3 elicited by violations contributes to the subsequent memory of events that violate these predictions.

At the retrieval stage, behavioral and neural research suggests two distinct processes contributing to memory recognition: familiarity and recollection (Jacoby 1991; Mandler 1980; Yonelinas 2002; for a recent review, Cowell, Barense, and Sadil 2019). While recollection has been defined traditionally as the assessment of specific details of an episode, a more nuanced perspective has arisen in recent decades. It has been proposed that recollection should be conceptualized not merely as the retrieval of specific details, as these details can sometimes be accessed through various cognitive processes (e.g., familiarity, Addante, Ranganath, and Yonelinas 2012). Rather, recollection should be viewed as the retrieval of an item linked to the contextual information from its previous episode (Diana, Yonelinas, and Ranganath 2007). This highlights the intricate nature of memory processes, underscoring that familiarity process, which is characterized as the subjective feeling that an event has been experienced before but in the absence of additional mnemonic details, can also contribute recognition. ERP studies have shown that recollection‐based memory is associated with a late parietal effect called late positive component (LPC), while the familiarity‐based memory is observed at frontal sites with an earlier time window referred as FN400 (Curran and Cleary 2003; Friedman 2013; Ozubko et al. 2021; Rugg and Curran 2007; Staresina and Wimber 2019). As previously mentioned, behaviorally, the effects of PEs on memory have been shown to extend to the retrieval phase, with differences between behavioral measures of familiarity and recollection (Kafkas and Montaldi 2018). However, neural evidence underlying these differences is limited (cf. McClure, Berns, and Montague 2003; Wittmann et al. 2007).

To gain a deeper understanding of how PEs influence the encoding and retrieval processes and their impact on episodic memory, we investigated the relationship between PEs, its potentially associated ERP components, and memory within a single paradigm. We employed a statistical learning paradigm, whereby participants implicitly learned sequentially presented object pairs embedded within a stream of objects over two consecutive days (Bein, Plotkin, and Davachi 2021). On the third day, new objects were added to the list. Half of the new objects were inserted in place of the second item of the pair, inducing PEs (violation items). The other half was presented between pairs, serving as a non‐violation baseline. Subsequently, participants' memory was assessed. We recorded electroencephalography (EEG) during encoding and retrieval phases.

We expected to replicate previous behavioral findings (Bein, Plotkin, and Davachi 2021), which demonstrated better memory performance for events that elicit PE compared to events that did not violate predictions. Additionally, we hypothesized that violating events that were later remembered would elicit larger P3 amplitudes compared to violating events that were later forgotten. Inspired by previous behavioral research (Kafkas and Montaldi 2018), we hypothesized that during retrieval, LPC would be observed for previously violated trials that were remembered, while FN400 would be observed for non‐violation trials that were remembered.

## 2 Method

### 2.1 Participants

51 university students (32 women, 13 men, mean age 23.52 [SD = 2.67]) were recruited for the study. A target sample size of 40 participants was determined by a power analysis of generalized linear mixed models (Green and MacLeod 2016) on our pilot data from 13 participants, which was not part of the final sample. The model was calculated with maximum‐likelihood estimation and participants as random intercept to account for between‐participant variability in the P3 mean amplitude during the violation phase. As fixed factors, we included the within‐participant factor of condition (violation and non‐violation) and item recognition accuracy (later remembered and later forgotten). The effect size for the interaction between condition and item recognition accuracy obtained from the pilot participants was 0.28. We accounted for potential effect size inflation by taking 90% of the effect size. Thus, we aimed to detect an effect size of 0.25 with the standard 0.05 alpha error to obtain 80% power. The pilot data and analysis scripts can be found on the study's OSF page (https://osf.io/sbc7d/).

Participants were recruited through an online experiment scheduling system of Goethe‐University Frankfurt am Main and personal contacts. All participants reported normal or corrected‐to‐normal vision, no neurological or psychiatric disorders, and right‐handedness. They were asked to sign informed consent approved by the local ethics committee of the Goethe‐University Frankfurt prior to the study, debriefed at the end, and compensated either with 10 € per hour or partial course credits.

Since the primary objective of the study was to examine the effects of violation on memory performance, we set two main exclusion criteria to ensure a clear interpretation of the results. In accordance with our pre‐registered plan, we excluded six participants with poor associative memory performance of < 40% accuracy rate and who showed poor recognition memory performance, meaning *d*’ below 0.35. The threshold for the *d*’ was calculated as that, it falls below 95% of the observations in a random distribution of *d*’ values after permuting the trial labels 5000 times, considering 100 old and 50 new trials in the memory test. Further details and code for calculating this threshold are available at the provided link (https://github.com/FPupillo/dprimethres). For associative memory performance, we set and pre‐registered a threshold of 40% accuracy, slightly above the chance level of 33%. This threshold was chosen to balance excluding participants with very low performance while ensuring that participants' performance reflected genuine learning. This threshold also aimed to maintain an adequate signal‐to‐noise ratio and include a sufficient number of participants for meaningful analysis. Additionally, four participants were excluded from further analysis steps due to missing or noisy EEG data. We ran the statistical analysis on the remaining 35 participants (26 women and 9 men, mean age 23.26, SD = 3.24).

### 2.2 Material

The stimulus set consisted of 370 pictures of everyday namable objects from the database used in the previous study (Bein, Plotkin, and Davachi 2021). The set was altered only in a few instances, where a picture of an object that may not be common in Germany was replaced with another object picture. The objects were presented with a white square background sized set to 350 × 350 pixels. The images were equally divided into two main categories according to their real‐life size based on whether they are bigger than a shoe box or not.

### 2.3 General Procedure

**FIGURE 1:**
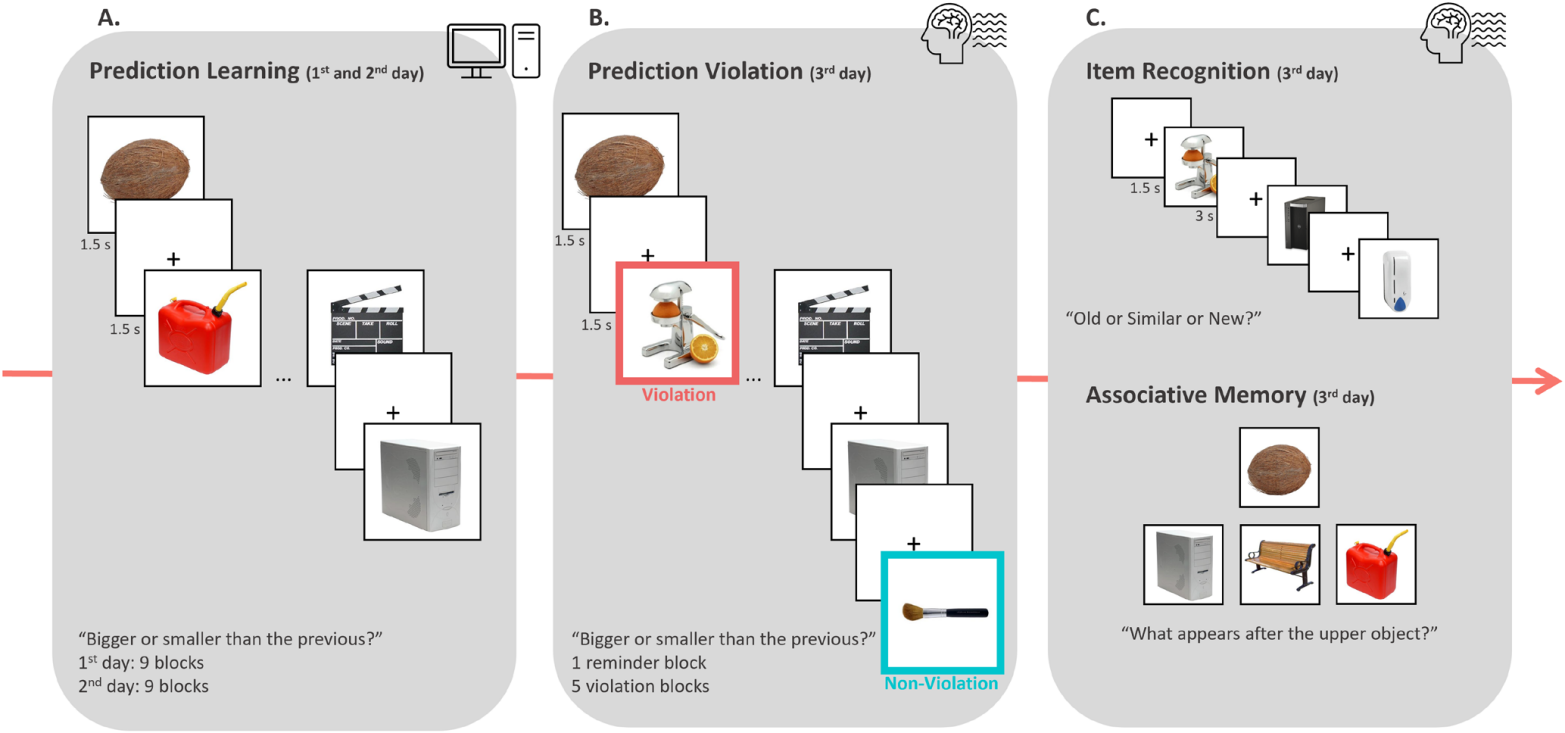
Study design. (A) During prediction learning (Days 1 and 2), participants viewed pairs of sequentially presented objects and were asked to indicate whether each object was bigger or smaller than the previous object. (B) In the prediction violation phase (Day 3), new object pictures were inserted into the sequence of objects, either instead of the second object in the pair (violation) or after the second object in a pair (non‐violation). (C) Following the violation phase, participants completed an item recognition memory test (Day 3) where they were presented with violation and non‐violation targets, similar lures, or new items, and asked to indicate whether each item was old, similar, or new. Memory for the original predictive pair was also tested (associative memory) by presenting participants with the first object in a pair and asking them to identify which of three objects followed the top object.

The study was conducted over three consecutive days (Figure 1). On the first 2 days, prediction learning phase took place and violation and retrieval phases were employed on the third day. Participants were presented with object pictures and asked to indicate if the presented object was bigger or smaller than the previous one. However, unbeknownst to the participants, there were pairs of objects that always followed each other, while the order of the pairs was randomized in each block. Participants who did not demonstrate signs of learning the pairs during the statistical learning phase were not invited to the third session, as they would not engage in prediction violation phase and therefore not experience PE. Thus, based on participants' response times (RTs) and accuracy rates on the bigger and smaller task, we decided if they were eligible to participate on the third day. We invited participants with RT differences of more than 200 ms between the first and second items across pairs and with accuracy rate more than 90% (*n* = 45). The third day started with a reminder, which included one block identical to the learning phase. Then, during the prediction violation phase, half of the original pairs were violated by replacing the second item in the pair with a new item. The other half remained intact and was followed by a new item to create a non‐violation baseline. Participants were then tested on surprise item recognition memory and associative memory, with a distraction task before and after the recognition memory phase.

To ensure participants' comfort and attention, we divided each task into multiple blocks and advised taking breaks in between. All instructions were provided both verbally and in written form. We used PsychoPy v2021.1.4 (Peirce 2007) to program the stimulus presentation and response collection. Each session was scheduled approximately 24 hours apart (*M* = 23.67, SD = 1.16).

#### 2.3.1 Prediction Learning Phase

The prediction learning phase used a statistical learning paradigm to build‐up predictions about object pairs. Participants implicitly learned sequentially presented object pairs embedded within a stream of objects over two consecutive days (Kim, Norman, and Turk‐Browne 2017; Schapiro, Kustner, and Turk‐Browne 2012; Turk‐Browne, Simon, and Sederberg 2012). Unbeknownst to the participants, there were object pairs that always followed each other, while the order of the pairs was randomized in each block. Each pair consisted of a big and a small object. Half of the pairs were presented with the big object first and the other half with the small object first. The pairing of objects was randomized for each participant while ensuring that each pair included one big and one small object.

During the task, participants were presented with a stream of object pictures and asked to indicate if the presented object was bigger or smaller than the previous one. Each trial started with a fixation cross at the center of the screen for 1.5 s and was followed by the object picture for 1.5 s. Participants were then asked to give a response by pressing C or M keys on the keyboard with their left or right index fingers. They were instructed to be as fast and as accurate as possible. Despite all object pictures on the screen appearing to be relatively the same size, they should base their judgments on the real‐life sizes of the objects. Before the initial task, participants received detailed instructions and completed eight practice trials. For each day, all participants completed 200 trials (100 pairs) equally spread over nine blocks.

#### 2.3.2 Reminder Phase

The third session of the study took place in the EEG laboratory. It started with a reminder phase, which was constructed as a block of the prediction learning phase. Participants covered one block of the previously presented 100 original pairs, in total 200 trials.

#### 2.3.3 Prediction Violation Phase

Immediately after the reminder phase, participants were presented with the prediction violation phase. The task structure was the same as the previous phases (i.e., prediction learning and reminder phases) and participants were not provided with additional instructions. Therefore, they were not explicitly informed of the transition to the prediction violation phase. In order to violate the pair associations, we added new object pictures to the list. For the violation condition, half of the original pairs (i.e., 50) were violated by replacing the second item in the pair with a new object picture. The other half of the original pairs (i.e., 50) remained intact and were followed by a new object picture to generate the non‐violation condition as a baseline. Only the identity of the item was violated but not the response, meaning that we replaced previously presented small objects with small new object pictures, and likewise big object pictures. Within each block, all pairs were presented twice. First, the original pairs were presented. For the second presentation, half of the pairs were violated, while the other half remained intact. There were 20 original pairs in each block. The presentation of the original, violation, and non‐violation pairs was randomized, with the constraint that there were at least six pairs between an original pair and its subsequent appearance as a violation or non‐violation pair. The prediction violation phase consisted of five blocks of 90 trials, for a total of 450 trials.

#### 2.3.4 Distraction Phase

Before and after the Item Recognition Phase (see below), participants performed a distraction task for 3 min in which a mix of basic mathematical operations, ranging in complexity were presented together with three alternative forced choices (e.g., 128–612 = ? and 8 × 5–507 = ?). In each trial, an equation was presented in the center of the screen and three response options appeared below. Participants used the “A,” “S,” or “D” keys on the keyboard to select the correct response and responded with their left hand's ring, middle or index finger. The respective letters were displayed under the response options to indicate the response keys. Once participants responded, the equation disappeared, and a new one appeared after a 500 ms delay. We informed participants to be as fast and accurate as possible.

#### 2.3.5 Item Recognition Phase

To test item recognition memory for both violation and non‐violation items, participants were presented with object pictures and were asked to indicate if the presented object was old, similar, or new. A total of 170 items were presented with 80 being identical to the items presented during the violation phase (half of the items were violation items and the other half was non‐violation items), 20 being similar lures that were different exemplars of objects presented during the violation phase, 20 being similar lures to the objects just seen in the recognition phase. In addition to violation items, non‐violation items, and similar lures, 50 new object pictures were also included. Our main focus was on the old trials. For that reason, we added similar lures to execute the task, while maximizing the number of old trials we could use for analysis. It should be noted that these lures were incorporated into the study to create a more challenging and sensitive recognition memory task for participants, inspired by previous findings (Bein, Plotkin, and Davachi 2021) showing that violations of expectations during learning could enhance memory for items' details. While similar lures were part of the study design, they were not the main focus, hence the low number of trials (i.e., 10 trials for each condition). Nevertheless, they played a role in calculating the classification index, a key measure in our study. The similar objects presented during the item recognition phase were defined such that participants were instructed to respond “old” if the object was identical to one presented during the previous phase, “similar” if it was a different exemplar of an object presented earlier, and “new” if it had not been presented before.

Each trial started with a fixation cross at the center of the screen for 1.5 s and was followed by the object picture for 3 s. To give a response, participants were instructed to press left, right, or down arrow keys on the keyboard with their ring, middle, or index finger of the right hand. The mapping of the left and right arrow key to indicate “old” or “new” responses was counterbalanced, while the down arrow key was consistently used for “similar” responses. Participants were clearly instructed to respond with “old” if the object was the same as an object presented during the previous phase, “similar” if the object was presented before, but it was not the exact object in the previous phase (i.e., a different exemplar), and “new” if the object was not presented before. For instance, if participants were initially shown a white computer during the prediction learning phase, a similar lure in the recognition phase might involve a black computer. During the task, there were indicators to show participants which key to use for each response, which disappeared once response was made. They started with a practice phase consisting of 12 trials via detailed instructions from the experimenter. All participants completed 170 trials equally spread over two blocks.

#### 2.3.6 Associative Memory Phase

After the item recognition phase, participants were given the second distraction task to reduce potential interference between the two memory phases. This was followed by an associative memory test, in which we aimed to assess explicit memory of the original pairs which were studied during the first two sessions of the study (i.e., prediction learning phase). Participants were instructed to indicate which object appeared after the top object during the initial two sessions. At the beginning of each trial, a fixation cross appeared at the upper center of the screen for 1.5 s. The first item of a pair was then presented at the upper center of the screen, accompanied by three alternative items located at the lower part of the screen. One of the three alternatives was the second item that corresponded to the first item of the original pair (i.e., target item). The other two alternatives were chosen from the second items that belonged to the same size category as the target item. Participants were asked to indicate which object appears after the upper object by pressing “A,” “S,” or “D” keys on the keyboard with their left hand's ring, middle, or index finger. Indicators were presented during the task to guide participants on which key to press for each object response. These indicators disappeared once the participant had made a response. In total, 100 trials were tested in one block after a practice phase of eight trials.

### 2.4 EEG Recording and Preprocessing

EEG was recorded during the third day of the study with 64 Ag/AgCI BrainProducts active electrodes (actiCAP; Brainproducts, Munich, Germany) following the international 10–10 system at Fp1, Fpz, Fp2, AF7, AF3, AF4, AF8, F7, F5, F3, F1, Fz, F2, F4, F6, F8, FT7, FC3, FC1, FC2, FC4, FT8, T7, C5, C3, C1, Cz, C2, C4, C6, T8, TP7, CP5, CP3, CP1, CPz, CP2, CP4, CP6, TP8, PO9, P7, P5, P3, P1, Pz, P2, P4, P6, P8, PO10, PO7, PO3, POz, PO4, PO8, O1, Oz, and O2 electrodes with a sampling rate of 1000 Hz (actiCHamp Plus amplifier; Brainproducts, Munich, Germany), online band‐pass filtered between 0 and 100 Hz. EEG data were online referenced to the left mastoid and a common ground was placed at the FCz. To record eye movements, three additional electrodes were placed at the outer canthi (horizontal electrooculography, EOG) and below the left eye (vertical EOG). Electrode impedance values were maintained below 20 kΩ during the recording.

EEG data preprocessing was performed offline with custom scripts in MNE‐Python 1.3 (Gramfort et al. 2014). It was run for each participant separately. As the first step, data were re‐referenced to both mastoid electrodes. Then, an independent component analysis was applied to correct eye blinks on cropped (we shortened the raw data to make it more manageable, thus reducing the computer memory required for the ICA decomposition process) and high pass (i.e., 1 Hz) filtered data. Those components were then corrected in three steps on the raw data: Automatic detection, visual check, and correction. Hereafter, data epochs were extracted according to the stimulus‐locked experimental conditions 100 ms prior to the onset of the stimuli presentation through 1500 ms post‐stimuli. We excluded the epochs containing values higher than 60 μV. The Autoreject function (Jas et al. 2017) was used to detect, interpolate, and reject bad epochs. Lastly, baseline corrected data were filtered between 0.1 and 30 Hz. After preprocessing, the mean total number of violation trials was 47.49 (SD = 2.89, range between 38 and 49) and non‐violation trials was 47.83 (SD = 2.37, range between 36 and 50) during the violation phase. For the recognition phase, the mean total number of remembered violation trials was 24.17 (SD = 2.41, range between 16 and 30), forgotten violation trials was 12.54 (SD = 2.67, range between 5 and 20), remembered non‐violation trials was 25.4 (SD = 2.29, range between 18 and 32) and forgotten non‐violation trials was 10.86 (SD = 2.13, range between 4 and 18).

### 2.5 Behavioral Analyses

As the first step, we calculated participants' “old” response rates to violation and non‐violation items, following Bein, Plotkin, and Davachi (2021) to compare our results. This was done only for the items for which the original pair was remembered correctly in the associative memory task. Second, we calculated classification indices based on confusion matrices (Ngo et al. 2021) to capture mnemonic discrimination. These classification indices are more sensitive than the traditional signal detection measures such as d’ and receiver operating characteristic curves, specifically in distinguishing old items from other categories such as similar and new items. To calculate classification indices, we first calculated the precision and sensitivity of violation and non‐violation items, each separately. Precision was computed as the ratio of correct old responses to all old responses, while sensitivity was calculated as the ratio of correct old responses to all old items. A classification index was then determined by multiplying precision and sensitivity by two, adding them together, and then dividing by the sum of precision and sensitivity (Ngo et al. 2021). Thus, the classification index takes into account not only the correctness of identifying old items but also the capacity to differentiate old items from other categories.

We then conducted general linear mixed‐effect model analyses for response rates and classification indices to investigate whether violation was a significant predictor of memory performance. All analyses were conducted with custom‐made R scripts (lme4 package: Bates et al. 2015) and can be found on the study's OSF page (https://osf.io/sbc7d/). The models included participants and objects as random intercepts, violation as fixed effect and random slope. We used a backward model selection approach. In this method, we ranked all possible models based on the number of parameters included in each one (Barr 2013). Starting from the full model, we compared the explanatory power of each model for the random effects via likelihood ratio test. We reduced the fixed effects by removing non‐significant predictors and interactions, and then compared these reduced models. Maximum likelihood ratio was assessed for model estimations and *χ* ^2^ (chi‐squared) was used for the statistical significance of the fixed effects. The model comparisons repeated until a significant decrease was observed. We also compared the models using AIC (Akaike Information Criterion) and BIC (Bayesian information criterion). An analysis of variance function was conducted to compare the variance explained by the models in terms of their model fit and to determine if the inclusion of random slopes for violation condition significantly improved the model. In the case of a significant interaction effect, we used the emmeans function to calculate estimated marginal means and performed post hoc tests with Bonferroni adjustment to compare the levels of predictors for each level of the other variable.

### 2.6 ERP Analyses

To investigate electrophysiological correlates of PEs, we measured P3 mean amplitude values at parietal electrodes during the violation phase. The mean amplitude values were calculated for the LPC and FN400 during the item recognition phase. ERPs were time‐locked to the onset of the stimuli. We defined time windows and electrodes for each component differently. The time window for the P3 component was 400–800 ms at centroparietal electrodes (CP3, CP1, CPz, CP2, CP4, P3, P1, Pz, P2, and P4). The FN400 was obtained during 300–500 ms after stimulus onset at frontocentral (F3, F1, Fz, F2, F4, F3, FC1, FC2, FC3, and FC4) electrodes. Lastly, the LPC was measured from 400 to 800 ms at parietooccipital (P3, P1, Pz, P2, P4, PO3, POz, and PO4) electrodes. The electrode selection was based on established ERP literature (Friedman 2013; Rugg and Curran 2007; Ozubko et al. 2021; Addante, Ranganath, and Yonelinas 2012). This selection was made to align with prior research and ensure consistency in our approach.

As suggested by Frömer, Maier, and Abdel Rahman (2018), linear mixed effect models were used to analyze trial‐based data with lmer function (lme4 package: Bates et al. 2015). The participants' mean‐centered amplitude values were introduced as dependent variables and modeled separately for P3, familiarity, and recollection components. The model included violation condition (violation vs. non‐violation), correct answer (correct vs. incorrect), and their interaction as fixed effects. The model also accounted for random effects by including random intercepts for participants and objects. Random slopes were not included in this model. As in the behavioral analyses, we follow the same rationale to test model comparisons for the random and interaction effects.

We also exploratorily used spatiotemporal cluster‐based permutation *t*‐tests (CBPT) to check the time window and topographical distributions. We created 3D data with channels, time points, and trials by participants for all scalp electrodes. Clusters were created by grouping adjacent channels and time points where the *p*‐values were lower than 0.05. The sum of all *t*‐values within a cluster was used to detect the following test statistic. This involved randomly assigning the samples into two classes and contrasting the differences between these random classes with the actual differences between our experimental conditions (e.g., violation vs. non‐violation trials for the prediction violation phase). This process was repeated 10,000 times for each permutation. Later, *t*‐statistics were calculated for each permutation and *t*‐values were summed for each cluster. All analyses were run with custom MNE‐Python scripts (Gramfort et al. 2014) and can be found on the study's OSF page (https://osf.io/sbc7d/).

### 2.7 Deviations From the Pre‐Registered Plan

The current study was preregistered prior to the data collection (https://osf.io/68jkz). All analyses were in line with our pre‐registered analysis plan, except that, in addition to d’ measure, we have also included the classification index, as it has demonstrated greater sensitivity (Ngo et al. 2021).

## 3 Results

### 3.1 Behavioral Results

Before conducting our primary analysis on the response rates and classification index, we first checked if participants learned the object pairs to build up predictions. Thus, we investigated the results from prediction learning, reminder, and associative memory phases. The RTs during the prediction learning and reminder phase were faster for the second item of the pair (*M* = 0.56, SD = 0.10) than the first item of the pair (*M* = 0.72, SD = 0.15), *t*(1, 38) = 60.97, *p* < 0.001, *d* = 0.38, indicating a learning process due to prediction of the upcoming object (see Appendix A). The accuracy rate during the associative memory phase to test original pairs was 0.78 (SD = 0.17) and at the group level, all participants selected the associated pair significantly above chance level, *t*(38) = 39.20, *p* < 0.001, *d* = 4.44. The accuracy rate for the original pairs was not different between violation and non‐violation trials, *t*(38) = −0.01, *p* = 0.99.

**FIGURE 2:**
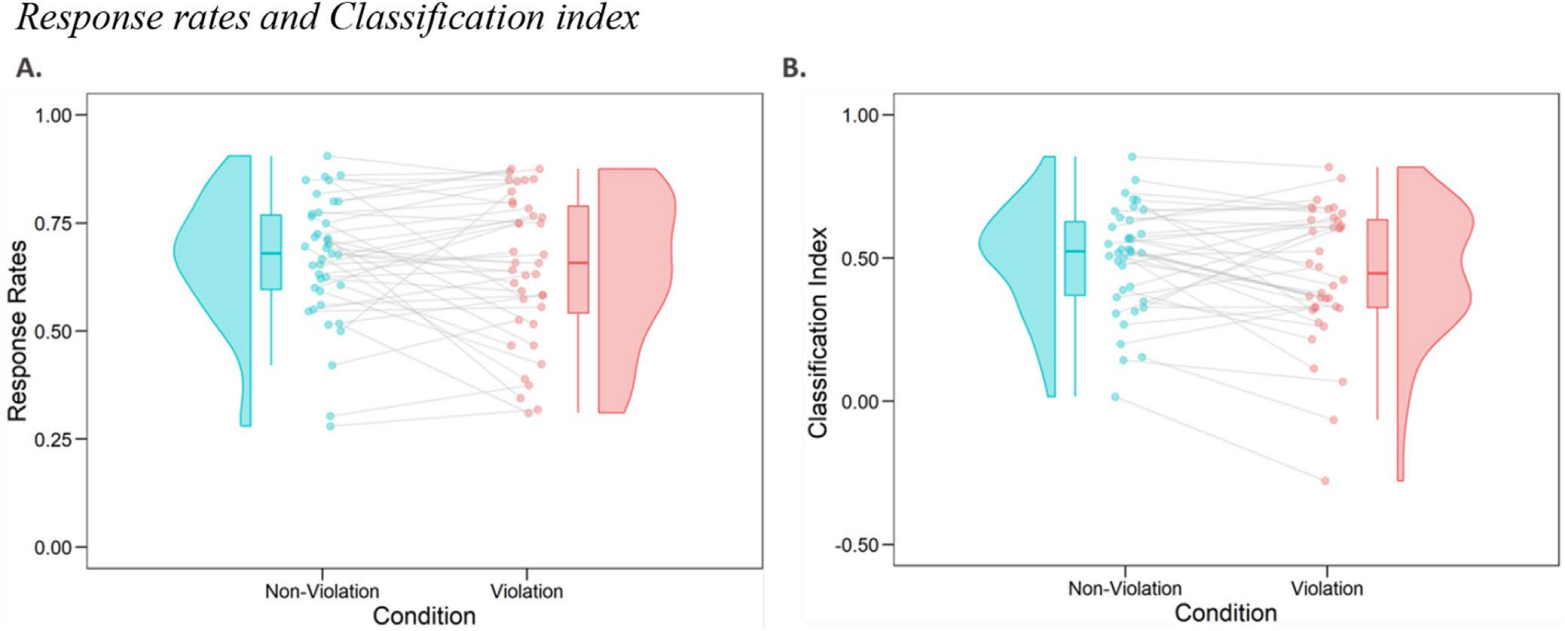
Response rates and classification index. The raincloud plot shows the distribution of response rates and classification index for violation and non‐violation conditions. (A) Proportion of old responses to old items. (B) The proportion of correct responses (true positives and true negatives) out of all instances. The box plots display the median, interquartile range, and 95% confidence interval for each group, while the density plots show the distribution of the data points for each experimental condition. The individual data points are displayed as scatter plots.

For the effects of PE on item recognition memory performance, the response rates and classification index are displayed in Figure 2. First, the full model to test the effect of violation condition on response rates did not show a significant main effect, *χ* ^*2*^(1) = 0.39, p = 0.53, and the full model did not significantly different from the reduced model without violation as a predictor, Δ*χ* ^2^(2) = 3.39, *p* = 0.18. Bayes Factors (BFs, Schönbrodt and Wagenmakers 2018) to index the evidence for the alternative hypothesis relative to the null hypothesis indicated moderate evidence for the null hypothesis (95% CI [−0.007, 0.133], BF01 = 5.15). Second, the classification index did not differ between violation items (*M* = 0.45, SD = 0.24) and non‐violation items (*M* = 0.49, SD = 0.19), *χ* ^2^(1) = 3.50, *p* = 0.06, 95% CI [−0.595, 0.035], BF01 = 1.23. Additionally, the participants' correct responses to similar lures did not differ between violation (*M* = 0.31, SD = 0.47) and non‐violation items (*M* = 0.32, SD = 0.47), *t*(1.38) = 0.38, *p* = 0.70, 95% CI [−0.512, 0.108], BF01 = 2.53. Alltogether, these findings indicate that there was no significant difference in item recognition memory performance between violation and non‐violation trials^1^ ^,^ ^2^. Although we did not find a behavioral difference in response rates and classification index, we proceeded to investigate our main hypotheses concerning ERP components as they could give better insights into mechanisms involved in encoding and retrieval processes of PE.

### 3.2 ERP Results

#### 3.2.1 P3 Amplitude During Encoding

**FIGURE 3:**
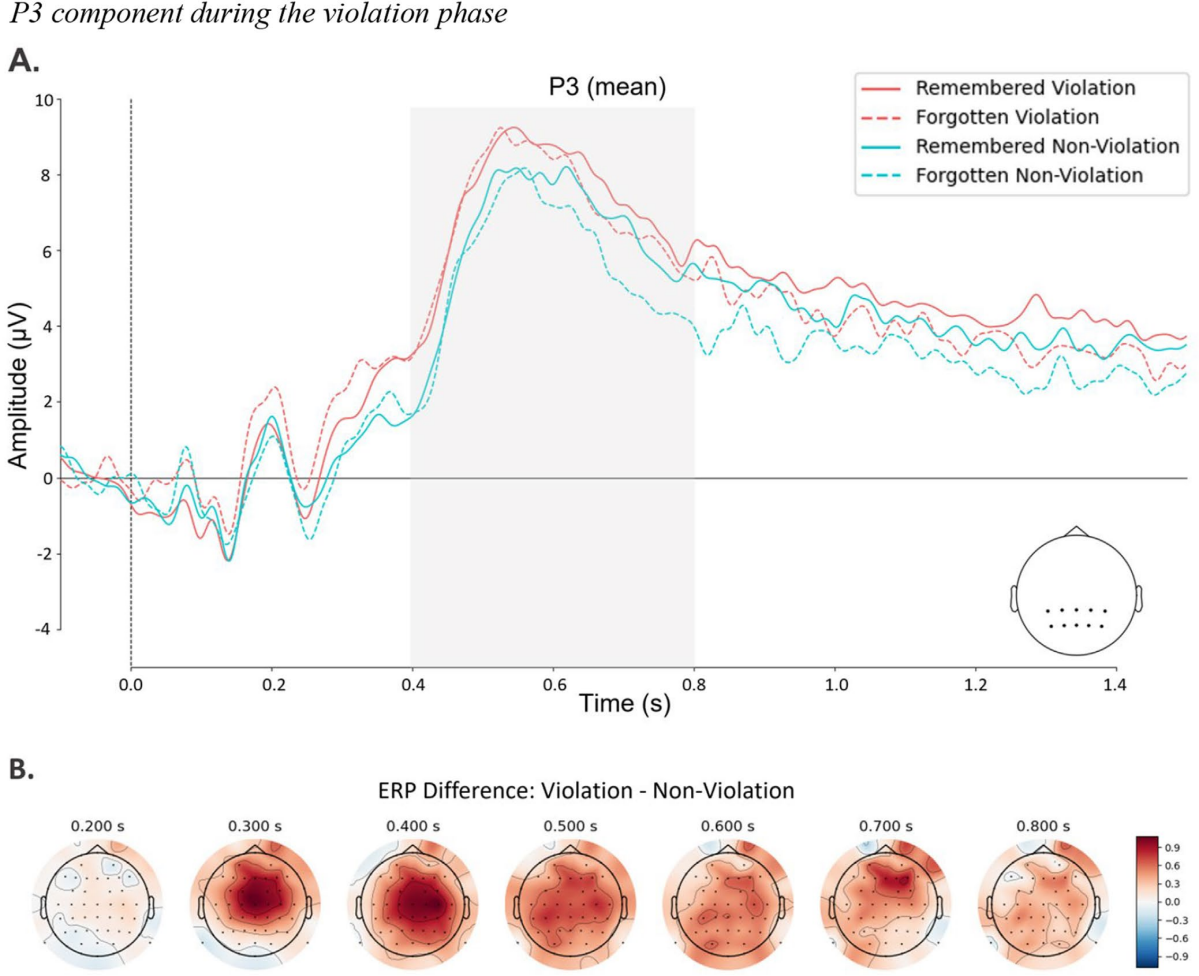
P3 component during the violation phase. Stimulus‐locked ERPs during the prediction violation phase. (A) Color‐coded ERP grand average recorded at centroparietal electrodes with highlighted time window in gray. (B) Topographical map plot of violation minus non‐violation difference in the P3 time window.

**FIGURE 4:**
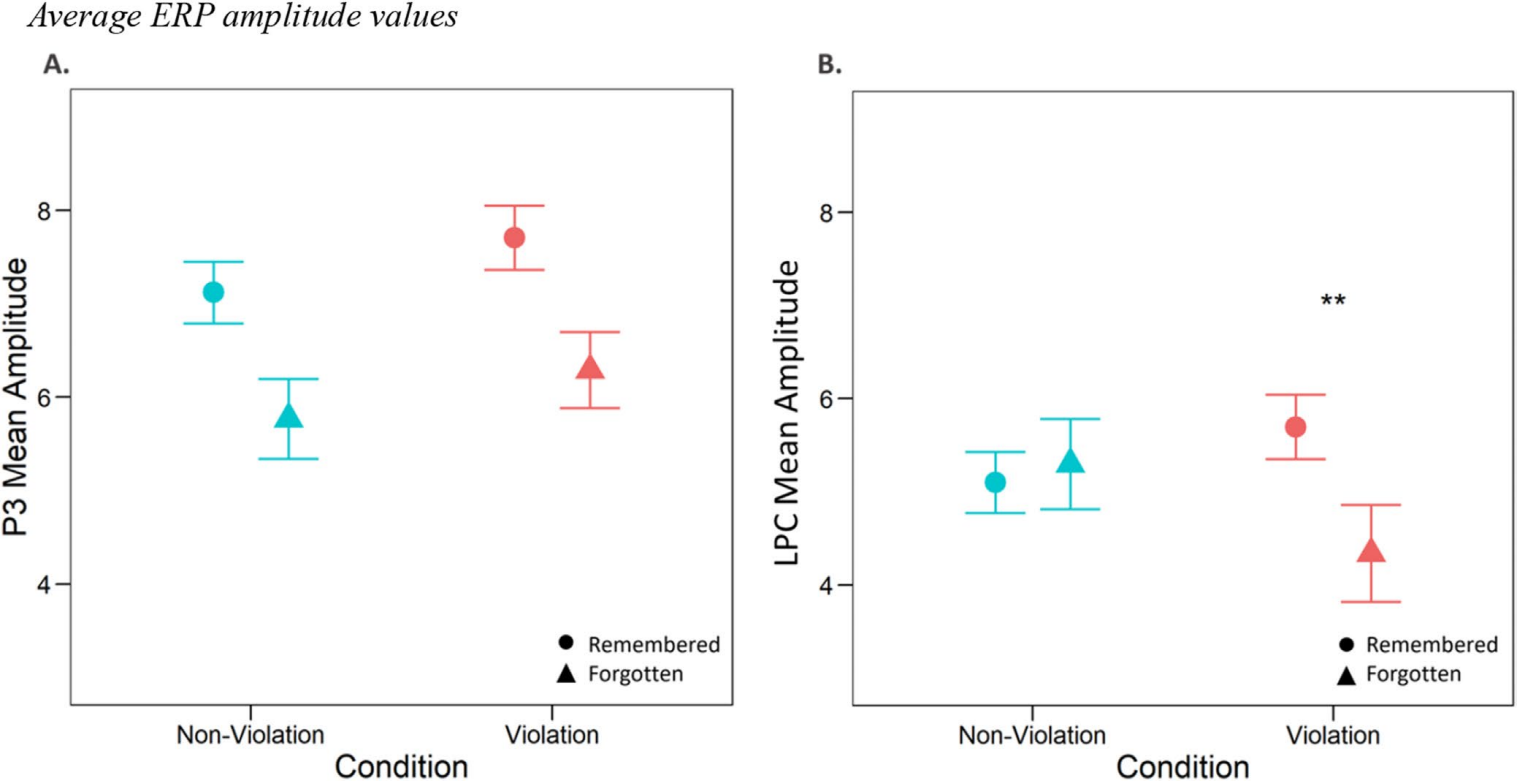
Average ERP amplitude values. Average ERP amplitude values for each condition within the relevant component‐specific time windows. Error bars represent the within‐participant standard error of the mean. (A) Mean amplitude values of P3 component during the violation phase. (B) Mean amplitude values of LPC during the item recognition phase. **p < 0.01.

The ERP results for the P3 amplitude can be seen in Figure 3. The average mean amplitude values are displayed in Figure 4A. We ran the analysis with mean amplitude values measured at centroparietal electrodes within the time window of 400 and 800 ms. We started with the full model with participants and objects as random intercepts and random slopes together with random slopes for the predictors to examine how P3 amplitude is influenced by violation condition and item accuracy. Model comparison favored the reduced model excluding violation, item accuracy, and their interaction as random slopes, Δ*χ* ^*2*^(18) = 8.05, *p* = 0.98 (AIC: 23394 vs. 23,366, BIC: 235451 vs. 23,408). The reduced model showed that the main effect of violation condition (*χ* ^*2*^(1) = 2.59, *p* = 0.11), item accuracy (*χ* ^*2*^(1) = 3.24, *p* = 0.07), and the interaction effect (*χ* ^*2*^(1) = 0.08, *p* = 0.08) was not significant. BFs indicated that there is anecdotal evidence for the null hypothesis for violation, item accuracy, and the interaction effects on P3 amplitude, respectively (95% CI [−0.219, 0.981], BF01 = 2.60; 95% CI [−0.130, 1.026], BF01 = 2.19; 95% CI [−0.825, 0.645], BF01 = 0.46). CBPT to compare violation and non‐violation trials found a cluster from 260 ms after stimulus onset to 532 ms for 53 electrodes (see Appendix B).

#### 3.2.2 LPC Amplitude During Retrieval

**FIGURE 5:**
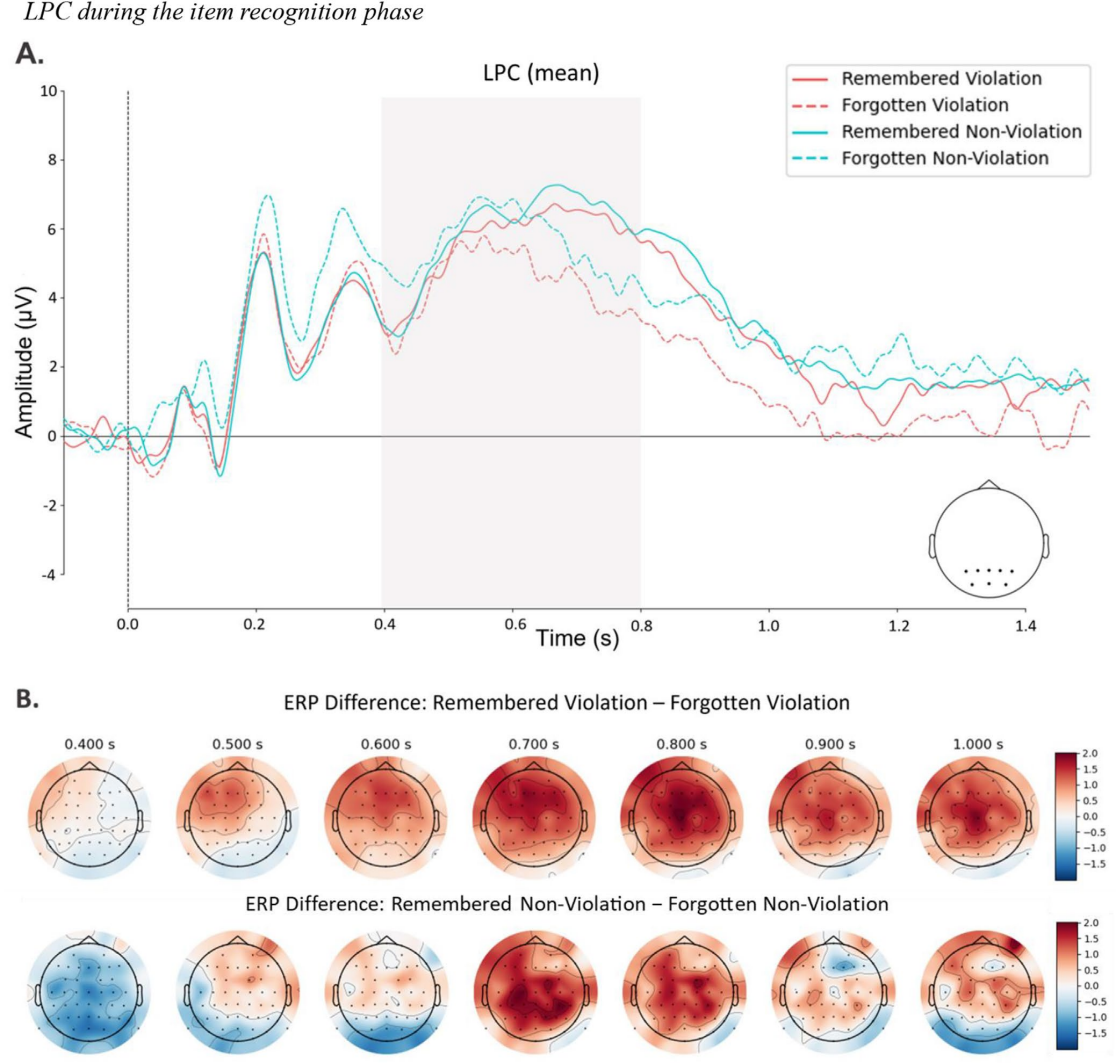
LPC during the item recognition phase. Stimulus‐locked ERPs during the item recognition phase. (A) Color‐coded ERP grand average recorded at parietooccipital electrodes with highlighted time window in gray. (B) Topographical map plot of remembered violation minus forgotten violation difference in the recollection component time window.

Figure 5 displays the ERP outcomes for the LPC. Figure 4B shows the average mean amplitude values for each condition. We conducted an analysis using mean amplitude values obtained at parietooccipital electrodes between 400 and 800 ms. We additionally focused on the 500–800 ms time window to provide a more detailed characterization of the LPC for its suggested later time frame (see Appendix D: Friedman 2013; Rugg and Curran 2007; Ozubko et al. 2021; Addante, Ranganath, and Yonelinas 2012). To test the effects of violation condition and item accuracy on the amplitude, we ran the full model with participants and trials as random intercepts and random slopes together with random slopes for the predictors. Model comparison favored the reduced model excluding violation, item accuracy, and their interaction as random slopes, Δ*χ* ^*2*^(18) = 12, *p* = 0.81 (AIC: 18445 vs. 18,421, BIC: 18591 vs. 18,462). In this reduced model without random slopes, the main effect of violation condition (*χ* ^2^(1) = 0.02, *p* = 0.88) and item accuracy (*χ* ^*2*^(1) = 2.55, *p* = 0.11) was not significant. However, the interaction of violation and item accuracy was significant, *χ* ^*2*^(1) = 3.87, *p* < 0.05. The follow‐up results showed that remembered violation trials had higher amplitudes than forgotten violation trials, *b* = 1.31, SE = 0.52, *p* = 0.01. There was no significant difference between remembered non‐violation and forgotten non‐violation trials, *b* = 0.15, SE = 0.54, *p* = 0.78.

Since we hypothesized that amplitude values during the late window of the item recognition phase would be higher for remembered violation items compared to forgotten violation items, suggesting a recollection effect, we conducted a CBPT only for remembered versus forgotten violation items. The results revealed a cluster between 524 and 1.177 ms after stimulus onset, involving 56 electrodes (see Appendix C). Additionally, we ran a CBPT only for non‐violation trials to test the effect of item memory, which found a cluster between 625 and 869 ms (see Appendix E).

#### 3.2.3 FN400 Amplitude During Retrieval

**FIGURE 6:**
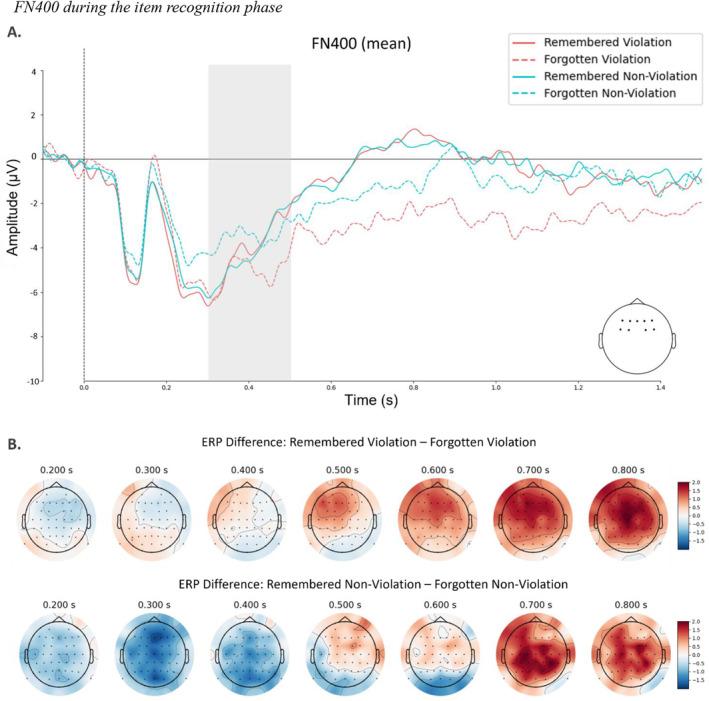
FN400 during the item recognition phase. Stimulus‐locked ERPs during the item recognition phase. (A) Color‐coded ERP grand average recorded at frontocentral electrodes with highlighted time window in gray. (B) Topographical map plot of remembered violation minus forgotten violation difference in the recollection component time window.

First, we conducted linear mixed effects models to investigate the effects of violation and item accuracy on the FN400 mean amplitudes obtained at frontocentral electrodes within 300 ms and 500 ms (Figure 6). Starting from the full model to the reduced model, there was no significant decrease in the model fit, Δ*χ* ^*2*^(18) = 8.20, *p* = 0.98. The main effects of violation, *χ* ^*2*^(1) = 0.66, *p* = 0.42, and item accuracy, *χ* ^2^(1) = 1.00, *p* = 0.32, and the interaction effect, *χ* ^*2*^(1) = 1.78, *p* = 0.18, were non‐significant. BFs provided anecdotal evidence for the null hypothesis for violation, item accuracy, and the interaction effects on FN400 amplitude (95% CI [−0.488, 0.810], BF01 = 5.81; 95% CI [−0.404, 0.926], BF01 = 3.70; 95% CI [−0.363, 1.339], BF01 = 2.03). CBPT analysis comparing the remembered and forgotten non‐violation trials did not find a cluster.

## 4 Discussion

The aim of this study was to investigate the electrophysiological correlates of encoding and retrieval of events eliciting PEs. To achieve this, we employed a statistical learning task, whereby participants implicitly learned pairs of objects. Subsequently, their memory was tested for predictions that were violated. Our behavioral results revealed successful learning of the object pairs. However, contrary to our pre‐registered hypothesis and prior findings (Bein, Plotkin, and Davachi 2021), we did not observe a memory advantage for items giving rise to PEs. Based on our ERP results, during retrieval, we found a significant association between the recollection component and item recognition memory for previously violated items. Specifically, there was a significant interaction, with higher amplitudes of the LPC for remembered violation trials compared to forgotten violation trials, but no difference between remembered and forgotten non‐violation trials. The results did not yield supporting evidence for the FN400. Furthermore, our data also did not show a link between P3 mean amplitude during encoding, PEs, and subsequent memory. Overall, these findings suggest that recollection influences the interplay between PE and episodic memory. Lastly, our exploratory analysis showed that our pre‐registered time windows for ERP components aligned with the cluster‐permutation results, indicating the validity of our approach in selecting relevant time‐windows of interest^3^.

In line with our pre‐registered hypothesis, we found higher amplitude values from 400 to 800 ms at parietooccipital electrodes for remembered violation trials compared to forgotten violation trials, indicating a recollection effect during retrieval of items that previously elicited PEs. This suggests that remembering events with PE might involve the retrieval of an item along with its associated contextual information from a previous episode. Notably, we observed a significant interaction effect, revealing a substantial difference in mean amplitudes of the LPC, specifically between remembered and forgotten violation items, but not within the non‐violation items. This finding suggests that the violation of expectations can enhance recollection, aligning with previous behavioral research demonstrating the retrieval‐enhancing effects of PEs (Kafkas and Montaldi 2018). The lack of a significant difference in the non‐violation condition implies that the mere presentation of baseline items may not be sufficient to enhance recollection. This could be because novel items, in the absence of a strong violation, fail to engage deeper levels of processing, such as processing and associating the episodic details of an event (Cowell, Barense, and Sadil 2019).

Our results regarding the LPC contribute to the growing body of evidence supporting the notion that memory‐guided predictions can enhance memory performance (Fenerci and Sheldon 2022; Henson and Gagnepain 2010; Theobald, Galeano‐Keiner, and Brod 2022; Van Kesteren et al. 2012). Memory‐guided predictions refer to the process by which retrieved memories of past events influence and shape predictions during the comprehension of unfolding events. For instance, Wahlheim et al. (2022) conducted a study investigating the effects of predictive‐looking errors on remembering event changes. Predictive‐looking errors occur when viewers direct their gaze to incorrect locations based on their memory of past experiences, but the actual event deviates from their predictions. In their study, participants watched movies of everyday activities, including actions that were repeated either identically or with changed features. Their findings demonstrated that memory guidance led to predictive‐looking errors, which were associated with better recollection memory for changed event features. This suggests that retrieving recent event features can guide predictions during unfolding events, and PEs can contribute to enhanced recollection when it is driven by expectations. In line with these findings, we observed a recollection process only for violation items, which were presented instead of the second object of the pairs that the participants had predicted to see. Taken together, our findings show that deviations from what was expected could generate a stronger recollection signal that facilitates subsequent memory.

However, in contrast to previous studies that have demonstrated better memory for events eliciting PEs (Antony et al. 2021; Bein, Plotkin, and Davachi 2021; Brod, Hasselhorn, and Bunge 2018; Greve et al. 2017; Quent, Greve, and Henson 2022), our study, despite utilizing a similar setup (Bein, Plotkin, and Davachi 2021), revealed a more nuanced pattern. We did not observe an overall memory advantage for PEs, but only differences in the neural correlates of retrieval of events that elicited PEs. The behavioral observation is consistent with recent studies that did not show memory‐enhancing effect of PEs (Ortiz‐Tudela et al. 2023; Turan et al. 2023). Thus, it is reasonable to consider that there may be additional factors moderating the relationship between PEs and subsequent memory benefit. Factors such as the strength and the precision of the prior (Greve et al. 2018; Ortiz‐Tudela et al. 2023), the appraisal (Gruber and Ranganath 2019) and the novelty (Schomaker and Meeter 2018) of the violation could potentially influence the effect of PEs on memory. In the following, we will discuss these factors and provide potential explanations for their presence in our results.

Our study protocol was similar to a previous study that demonstrated the beneficial effect of PEs on memory (Bein, Plotkin, and Davachi 2021). However, there was a main difference between our study and the study by Bein, Plotkin, and Davachi's (2021) which was the increased number of object pairs and blocks. To ensure an adequate signal‐to‐noise ratio for the EEG signal, we increased the number of trials from 36 to 50 for each condition, necessitating additional blocks and sessions to achieve an effective learning threshold. Consequently, our extended learning phase likely resulted in stronger predictions compared to the previous study (Bein, Plotkin, and Davachi 2021), where the reported accuracy rate was 0.60, whereas in our study, it was 0.78. As a result, our participants may have had stronger predictions, leading to higher item surprise for violation trials (Greve et al. 2017; Quent, Henson, and Greve 2021). It is reasonable to assume that stronger predictions are associated with higher PEs and that might have resulted in improved subsequent memory. However, according to a recent framework (PACE: Gruber and Ranganath 2019), the memory enhancement for PEs is not solely determined by prediction strength but also by appraisal. This framework proposes that PEs trigger an appraisal process that influences one's actions and subjective experience in resolving the uncertainty elicited by PEs. This process can either trigger curiosity and subsequent memory enhancement or elicit behavioral inhibition due to negative uncertainty assessment. In our study, participants may have exhibited a tendency to disregard the new objects altogether, violation and non‐violation objects presented during the violation phase, instead relied more heavily on the previously learned objects, possibly indicating a negative assessment of uncertainty resolution. Congruently, a similar finding was reported in one of our recent studies (Ortiz‐Tudela et al. 2023), which demonstrated a decreased memory performance for violations of strong predictions derived from low‐uncertainty priors.

Furthermore, the role of context surprise (Quent, Henson, and Greve 2021) should be taken into account when interpreting our findings. Our task involved extensive exposure to the paired structure of object associations, which could have created context surprise when participants encountered a non‐violation item that violated the expected task structure. Specifically, violation items violated the expected object at the item level, whereas non‐violation items violated the expected task structure by presenting an object that had not been previously seen in that specific position (i.e., after the second object of a pair), thereby creating a context surprise and leading to novelty. This distinction may have elicited different cognitive and neural responses compared to the violation items that violated the expected object at the item level. Therefore, the absence of a memory benefit for PEs and its relationship to the P3 component in our study could potentially be attributed to both experimental conditions engendering expectations and subsequent violations of those expectations (Schomaker and Meeter 2018). Additionally, since our task involved a statistical learning paradigm, we did not specifically measure participants' awareness of the violations, which could have influenced their responses and neural correlates. Future studies should consider assessing this awareness to better understand this complex relationship between PE and memory processes.

While our current study focused on the electrophysiological correlates that are not easily localized in the brain, to gain a comprehensive view of the neural mechanisms underlying the impact of PE on subsequent memory, it is important to consider anatomical regions underlying episodic memory. Prior research has shown that the hippocampus is involved in both familiarity and recollection (Merkow, Burke, and Kahana 2015). However, the temporal dynamics of these processes in the hippocampus differ. For example, an intracranial EEG study revealed distinctive hippocampal responses during successful memory retrieval emerging between 500 and 1500 ms post stimulus, indicating a recollection process (Staresina et al. 2012). This finding aligns with our LPC and CBPT findings, suggesting that a hippocampal signal that distinguishes successful from unsuccessful memory performance can be detected at later time points. On the other hand, in contrast to prior research that has reported a relationship between subsequent memory, P3, and hippocampal activity (Fonken, Kam, and Knight 2020), our study, similar to some others (Höltje and Mecklinger 2022; Rangel‐Gomez and Meeter 2013), did not demonstrate a direct link between expectancy, successful memory and the P3 component. Even though this discrepancy could be attributed to variations in the types of memory tests employed (Quent, Henson, and Greve 2021), it indicates that there may be more nuanced dynamics at play in this relationship. Future investigations can build upon our findings by incorporating neuroimaging techniques to pinpoint the specific brain regions associated with the encoding and retrieval processes of unexpected events (e.g., Bein, Reggev, and Maril 2020; Kumaran and Maguire 2006; Sinclair et al. 2021).

Our results highlight the importance of recollection as a potential mechanism underlying the association between PEs and episodic memory processes. Even though our findings indicate differences in recollection related to PE, we acknowledge that the absence of behavioral differences needs further explanation and investigation (Yacoby, Reggev, and Maril 2021). It raises questions about the robustness of an overall beneficial effect of PEs on episodic memory, while at the same time highlights the value of EEG in revealing subtle differences in memory processes. The null behavioral effects of PE on memory performance in our study may not imply an absence of PE. Instead, it suggests that other factors, such as the strength and the precision of priors, appraisal, and novelty of the violation (Greve et al. 2018; Gruber and Ranganath 2019; Ortiz‐Tudela et al. 2023; Schomaker and Meeter 2018), may moderate the relationship between PE and memory benefit. Future research should consider these factors to better understand this complex relationship.

In conclusion, our findings on higher LPC amplitudes for remembered violation trials compared to forgotten violation trials, with no difference observed for non‐violation trials, contribute to our understanding of how we remember unexpected events, when successfully done. However, not all events with PE are consistently remembered later on, as shown by a lack of overall better memory performance for events with violation. This aligns with the notion that while PE can generate neural signatures indicative for recollection, this may not always happen or translate into measurable behavioral outcomes. Further investigation into the recollection process could provide a more comprehensive understanding of how PEs influence memory. Overall, our study contributes to the growing body of knowledge on the complex and nuanced nature of the relationship between PE and episodic memory processes, shedding light on the underlying neural mechanisms involved.

## Author Contributions

**Gözem Turan:** conceptualization, data curation, formal analysis, investigation, methodology, project administration, visualization, writing – original draft, writing – review and editing. **Veronika Spiertz:** data curation, investigation, writing – review and editing. **Oded Bein:** conceptualization, methodology, resources, writing – review and editing. **Yee Lee Shing:** conceptualization, funding acquisition, methodology, project administration, supervision, writing – review and editing. **Sophie Nolden:** conceptualization, funding acquisition, methodology, project administration, supervision, writing – review and editing.

## Conflicts of Interest

The authors declare no conflicts of interest.

## Data Availability

Data, scripts, and additional online materials are openly available on the project's Open Science Framework page (https://osf.io/sbc7d/).

## Endnotes

^1^
In addition to response rates and classification index, we also calculated d’ scores as stated in the pre‐registered report. The results did not show a main effect of violation on d’ scores, either, *χ* ^*2*^(1) = 2.37, *p* = 0.12.

^2^
We also analyzed memory performance without filtering the data based on the associative memory performance. The effect of violation condition on response rates did not yield a significant main effect (Δ*χ* ^2^(2) = 2.64, *p* = 0.27), suggesting that the approach to analyze the item recognition did not change the overall results, namely that there was no difference between violation and non‐violation items and both sets of analyses align in their general findings.

^3^
The CBPT not only allowed us to identify significant effects but also served as a confirmatory tool, validating our choice of time windows for investigating the ERP components (Frömer, Maier, and Abdel Rahman 2018).

## Appendix A

### A.1

- RT differences between the first and second item of the pairs during the prediction learning and reminder phases.
- CBPT results for the P3 mean amplitude for the comparison violation and non‐violation trials.
- CBPT results for the recollection component for the comparison remembered violation and forgotten violation trials.
- The analysis for calculating mean amplitude values within the 500–800 ms time window at the parietooccipital electrodes.
- CBPT results for the recollection component for the comparison remembered non‐violation and forgotten non‐violation trials.

D. We conducted a similar linear mixed‐effects models approach to test the effects of violation condition and item accuracy on the LPC amplitude. Results from this extended analysis revealed that the model comparison favored the reduced model excluding violation, item accuracy, and their interaction as random slopes, *Δχ* ^2^(18) = 11.84, *p* = 0.81 (AIC: 18741 vs. 18,717, BIC: 18887 vs. 18,758). In this reduced model without random slopes, while the main effect of violation condition (*χ* ^2^(1) = 0.02, *p* = 0.90) was not significant, the item accuracy (*χ* ^2^(1) = 4.42, *p* < 0.05) was significant. We observed higher amplitude values for remembered trials than forgotten trials (*b* = 0.82, SE = 0.40). Additionally, the interaction of violation and item accuracy was significant, *χ* ^2^(1) = 3.67, *p* < 0.05. The follow‐up results showed that remembered violation trials had higher amplitudes than forgotten violation trials, *b* = 1.58, SE = 0.56, *p* < 0.01. There was no significant difference between remembered non‐violation and forgotten non‐violation trials, *b* = 0.06, SE = 0.58, *p* = 0.91.

## References

[psyp14752-bib-0001] Addante, R. J. , C. Ranganath , and A. P. Yonelinas . 2012. “Examining ERP Correlates of Recognition Memory: Evidence of Accurate Source Recognition Without Recollection.” NeuroImage 62, no. 1: 439–450. 10.1016/J.NEUROIMAGE.2012.04.031. 22548808 PMC3381051

[psyp14752-bib-0002] Aisa, B. , B. Mingus , and R. O'Reilly . 2008. “The Emergent Neural Modeling System.” Neural Networks 21: 1146–1152. 10.1016/j.neunet.2008.06.016. 18684591

[psyp14752-bib-0003] Aitchison, L. , and M. Lengyel . 2017. “With or Without You: Predictive Coding and Bayesian Inference in the Brain.” Current Opinion in Neurobiology 46: 219–227. 10.1016/J.CONB.2017.08.010. 28942084 PMC5836998

[psyp14752-bib-0004] Alba, J. W. , and L. Hasher . 1983. “Is Memory Schematic?” Psychological Bulletin 93, no. 2: 203–231. 10.1037/0033-2909.93.2.203.

[psyp14752-bib-0005] Alonso, A. , J. van der Meij , D. Tse , and L. Genzel . 2020. “Naïve to Expert: Considering the Role of Previous Knowledge in Memory.” Brain and Neuroscience Advances 2020, no. 4: 2398212820948686. 10.1177/2398212820948686. PMC7479862 32954007

[psyp14752-bib-0006] Antony, J. W. , T. H. Hartshorne , K. Pomeroy , et al. 2021. “Behavioral, Physiological, and Neural Signatures of Surprise During Naturalistic Sports Viewing.” Neuron 109, no. 2: 377–390. 10.1016/j.neuron.2020.10.029. 33242421

[psyp14752-bib-0007] Bar, M. 2007. “The Proactive Brain: Using Analogies and Associations to Generate Predictions.” Trends in Cognitive Sciences 11, no. 7: 280–289. 10.1016/j.tics.2007.05.005. 17548232

[psyp14752-bib-0008] Barr, D. J. 2013. “Random Effects Structure for Testing Interactions in Linear Mixed‐Effects Models.” Frontiers in Psychology 4, no. 1: 328. 10.3389/FPSYG.2013.00328. 23761778 PMC3672519

[psyp14752-bib-0009] Bates, D. , M. Mächler , B. M. Bolker , and S. C. Walker . 2015. “Fitting Linear Mixed‐Effects Models Using lme4.” Journal of Statistical Software 67, no. 1. 10.18637/jss.v06i01.

[psyp14752-bib-0010] Bein, O. , and L. Davachi . 2022. “Event Integration and Temporal Pattern Separation: How Hierarchical Knowledge Emerges in Hippocampal Subfields Through Learning.” 10.1101/2022.07.18.500527. PMC10919070 38129134

[psyp14752-bib-0011] Bein, O. , C. Gasser , T. Amer , A. Maril , and L. Davachi . 2023. “Predictions Transform Memories: How Expected versus Unexpected Events Are Integrated or Separated in Memory.” Neuroscience & Biobehavioral Reviews 153: 105368. 10.1016/J.NEUBIOREV.2023.105368. 37619645 PMC10591973

[psyp14752-bib-0012] Bein, O. , N. A. Plotkin , and L. Davachi . 2021. “Mnemonic Prediction Errors Promote Detailed Memories.” Learning and Memory 28, no. 11: 422–434. 10.1101/LM.053410.121. 34663695 PMC8525423

[psyp14752-bib-0013] Bein, O. , N. Reggev , and A. Maril . 2020. “Prior Knowledge Promotes Hippocampal Separation but Cortical Assimilation in the Left Inferior Frontal Gyrus.” Nature Communications 11, no. 1: 1–13. 10.1038/s41467-020-18364-1. PMC7490707 32929067

[psyp14752-bib-0014] Brod, G. , M. Hasselhorn , and S. A. Bunge . 2018. “When Generating a Prediction Boosts Learning: The Element of Surprise.” Learning and Instruction 55: 22–31. 10.1016/J.LEARNINSTRUC.2018.01.013.

[psyp14752-bib-0015] Brod, G. , and Y. L. Shing . 2019. “A Boon and a Bane: Comparing the Effects of Prior Knowledge on Memory Across the Lifespan.” Developmental Psychology 55, no. 6: 1326–1337. 10.1037/dev0000712. 30802088

[psyp14752-bib-0016] Cowell, R. A. , M. D. Barense , and P. S. Sadil . 2019. “A Roadmap for Understanding Memory: Decomposing Cognitive Processes Into Operations and Representations.” eNeuro 6, no. 4: 1–19. 10.1523/ENEURO.0122-19.2019. PMC6620388 31189554

[psyp14752-bib-0017] Craik, F. I. , and E. Tulving . 1975. “Depth of Processing and the Retention of Words in Episodic Memory.” Journal of Experimental Psychology: General 104, no. 3: 268–294. 10.1037/0096-3445.104.3.268.

[psyp14752-bib-0018] Curran, T. , and A. M. Cleary . 2003. “Using ERPs to Dissociate Recollection From Familiarity in Picture Recognition.” Cognitive Brain Research 15, no. 2: 191–205. 10.1016/S0926-6410(02)00192-1. 12429370

[psyp14752-bib-0019] Diana, R. A. , A. P. Yonelinas , and C. Ranganath . 2007. “Imaging Recollection and Familiarity in the Medial Temporal Lobe: A Three‐Component Model.” Trends in Cognitive Sciences 11, no. 9: 379–386. 10.1016/J.TICS.2007.08.001. 17707683

[psyp14752-bib-0020] Donchin, E. 1981. “Surprise!… Surprise?” Psychophysiology 18, no. 5: 493–513. 10.1111/j.1469-8986.1981.tb01815.x. 7280146

[psyp14752-bib-0021] Ergo, K. , E. De Loof , and T. Verguts . 2020. “Reward Prediction Error and Declarative Memory.” Trends in Cognitive Sciences 24, no. 5: 388–397. 10.1016/J.TICS.2020.02.009. 32298624

[psyp14752-bib-0022] Fabiani, M. , D. Karis , and E. Donchin . 1986. “P300 and Recall in an Incidental Memory Paradigm.” Psychophysiology 23, no. 3: 298–308. 10.1111/j.1469-8986.1986.tb00636.x. 3749410

[psyp14752-bib-0023] Fenerci, C. , and S. Sheldon . 2022. “The Role of Episodic Memory in Imagining Autobiographical Events: The Influence of Event Expectancy and Context Familiarity.” Memory 30, no. 5: 573–590. 10.1080/09658211.2022.2032178. 35129426

[psyp14752-bib-0024] Fonken, Y. M. , J. W. Y. Kam , and R. T. Knight . 2020. “A Differential Role for Human Hippocampus in Novelty and Contextual Processing: Implications for P300.” Psychophysiology 57, no. 7: e13400. 10.1111/PSYP.13400. 31206732

[psyp14752-bib-0025] Frank, D. , M. A. Montemurro , and D. Montaldi . 2020. “Pattern Separation Underpins Expectation‐Modulated Memory.” Journal of Neuroscience 40, no. 17: 3455–3464. 10.1523/JNEUROSCI.2047-19.2020. 32161140 PMC7178906

[psyp14752-bib-0026] Friedman, D. 2013. “The Cognitive Aging of Episodic Memory: A View Based on the Event‐Related Brain Potential.” Frontiers in Behavioral Neuroscience 7: 58053. 10.3389/FNBEH.2013.00111/BIBTEX. PMC3752587 23986668

[psyp14752-bib-0027] Friedman, D. , Y. M. Cycowicz , and H. Gaeta . 2001. “The Novelty P3: An Event‐Related Brain Potential (ERP) Sign of the Brain's Evaluation of Novelty.” Neuroscience & Biobehavioral Reviews 25, no. 4: 355–373. 10.1016/S0149-7634(01)00019-7. 11445140

[psyp14752-bib-0028] Friston, K. 2010. “The Free‐Energy Principle: A Unified Brain Theory?” Nature Reviews Neuroscience 11, no. 2: 127–138. 10.1038/nrn2787. 20068583

[psyp14752-bib-0029] Frömer, R. , M. Maier , and R. Abdel Rahman . 2018. “Group‐Level EEG‐Processing Pipeline for Flexible Single Trial‐Based Analyses Including Linear Mixed Models.” Frontiers in Neuroscience 12: 1–15. 10.3389/fnins.2018.00048. 29472836 PMC5810264

[psyp14752-bib-0030] Gramfort, A. , M. Luessi , E. Larson , et al. 2014. “MNE Software for Processing MEG and EEG Data.” NeuroImage 86: 446–460. 10.1016/j.neuroimage.2013.10.027. 24161808 PMC3930851

[psyp14752-bib-0031] Green, P. , and C. J. MacLeod . 2016. “SIMR: An R Package for Power Analysis of Generalized Linear Mixed Models by Simulation.” Methods in Ecology and Evolution 7, no. 4: 493–498. 10.1111/2041-210X.12504.

[psyp14752-bib-0032] Greve, A. , E. Cooper , A. Kaula , M. C. Anderson , and R. Henson . 2017. “Does Prediction Error Drive One‐Shot Declarative Learning?” Journal of Memory and Language 94: 149–165. 10.1016/j.jml.2016.11.001. 28579691 PMC5381756

[psyp14752-bib-0033] Greve, A. , E. Cooper , R. Tibon , and R. N. Henson . 2018. “Knowledge Is Power: Prior Knowledge Aids Memory for Both Congruent and Incongruent Events, but in Different Ways.” Journal of Experimental Psychology: General 148, no. 2: 325–341. 10.1037/XGE0000498. 30394766 PMC6390882

[psyp14752-bib-0034] Gruber, M. J. , and C. Ranganath . 2019. “How Curiosity Enhances Hippocampus‐Dependent Memory: The Prediction, Appraisal, Curiosity, and Exploration (PACE) Framework.” Trends in Cognitive Sciences 23, no. 12: 1014–1025. 10.1016/j.tics.2019.10.003. 31706791 PMC6891259

[psyp14752-bib-0035] Henson, R. N. , and P. Gagnepain . 2010. “Predictive, Interactive Multiple Memory Systems.” Hippocampus 20, no. 11: 1315–1326. 10.1002/hipo.20857. 20928831

[psyp14752-bib-0036] Höltje, G. , and A. Mecklinger . 2022. “Benefits and Costs of Predictive Processing: How Sentential Constraint and Word Expectedness Affect Memory Formation.” Brain Research 1788: 147942. 10.1016/J.BRAINRES.2022.147942. 35562077

[psyp14752-bib-0037] Jacoby, L. L. 1991. “A Process Dissociation Framework: Separating Automatic From Intentional Uses of Memory.” Journal of Memory and Language 30, no. 5: 513–541. 10.1016/0749-596X(91)90025-F.

[psyp14752-bib-0038] Jas, M. , D. A. Engemann , Y. Bekhti , F. Raimondo , and A. Gramfort . 2017. “Autoreject: Automated Artifact Rejection for MEG and EEG Data.” NeuroImage 159: 417–429. 10.1016/j.neuroimage.2017.06.030. 28645840 PMC7243972

[psyp14752-bib-0039] Kafkas, A. , and D. Montaldi . 2018. “Expectation Affects Learning and Modulates Memory Experience at Retrieval.” Cognition 180: 123–134. 10.1016/J.COGNITION.2018.07.010. 30053569 PMC6191926

[psyp14752-bib-0040] Kim, G. , K. A. Norman , and N. B. Turk‐Browne . 2017. “Neural Differentiation of Incorrectly Predicted Memories.” Journal of Neuroscience 37, no. 8: 2022–2031. 10.1523/JNEUROSCI.3272-16.2017. 28115478 PMC5338753

[psyp14752-bib-0041] Kramer, A. F. , C. D. Wickens , and E. Donchin . 1985. “Processing of Stimulus Properties: Evidence for Dual‐Task Integrality.” Journal of Experimental Psychology: Human Perception and Performance 11, no. 4: 393–408. 10.1037/0096-1523.11.4.393. 3161983

[psyp14752-bib-1001] Kumaran, D. , and E. A. Maguire . 2006. “An Unexpected Sequence of Events: Mismatch Detection in the Human Hippocampus.” PLoS Biology 4, no. 12: e424. 10.1371/journal.pbio.0040424. 17132050 PMC1661685

[psyp14752-bib-0042] Liu, Z. X. , C. Grady , and M. Moscovitch . 2018. “The Effect of Prior Knowledge on Post‐Encoding Brain Connectivity and Its Relation to Subsequent Memory.” NeuroImage 167: 211–223. 10.1016/J.NEUROIMAGE.2017.11.032. 29158201

[psyp14752-bib-0043] Lu, Q. , U. Hasson , and K. A. Norman . 2022. “A Neural Network Model of When to Retrieve and Encode Episodic Memories.” eLife 11: 1–43. 10.7554/ELIFE.74445. PMC9000961 35142289

[psyp14752-bib-0044] Mandler, G. 1980. “Recognizing: The Judgment of Previous Occurrence.” Psychological Review 87, no. 3: 252–271. 10.1037/0033-295X.87.3.252.

[psyp14752-bib-0045] McClure, S. M. , G. S. Berns , and P. R. Montague . 2003. “Temporal Prediction Errors in a Passive Learning Task Activate Human Striatum.” Neuron 38, no. 2: 339–346. 10.1016/S0896-6273(03)00154-5. 12718866

[psyp14752-bib-0046] Merkow, M. B. , J. F. Burke , and M. J. Kahana . 2015. “The Human Hippocampus Contributes to Both the Recollection and Familiarity Components of Recognition Memory.” Proceedings of the National Academy of Sciences 112, no. 46: 14378–14383. 10.1073/pnas.1513145112. PMC4655532 26578784

[psyp14752-bib-0047] Ngo, C. T. , S. Michelmann , I. R. Olson , and N. S. Newcombe . 2021. “Pattern Separation and Pattern Completion: Behaviorally Separable Processes?” Memory & Cognition 49: 193–205. 10.3758/s13421-020-01072-y. 32728851 PMC7819938

[psyp14752-bib-0048] Ortiz‐Tudela, J. , B. Milliken , F. Botta , M. LaPointe , and J. Lupiañez . 2017. “A Cow on the Prairie vs. a Cow on the Street: Long‐Term Consequences of Semantic Conflict on Episodic Encoding.” Psychological Research 81, no. 6: 1264–1275. 10.1007/s00426-016-0805-y. 27638300

[psyp14752-bib-0049] Ortiz‐Tudela, J. , S. Nolden , F. Pupillo , et al. 2023. “Not What u Expect: Effects of Prediction Errors on Item Memory.” Journal of Experimental Psychology: General 3: 2160–2176. 10.1037/XGE0001367. 36996155

[psyp14752-bib-0050] Ozubko, J. D. , L. A. Sirianni , F. N. Ahmad , C. M. MacLeod , and R. J. Addante . 2021. “Recallable but Not Recognizable: The Influence of Semantic Priming in Recall Paradigms.” Cognitive, Affective, & Behavioral Neuroscience 21, no. 1: 119–143. 10.3758/S13415-020-00854-W. PMC7994187 33409957

[psyp14752-bib-0051] Paz, R. , H. Gelbard‐Sagiv , R. Mukamel , M. Harel , R. Malach , and I. Fried . 2010. “A Neural Substrate in the Human Hippocampus for Linking Successive Events.” Proceedings of the National Academy of Sciences of the United States of America 107, no. 13: 6046–6051. 10.1073/pnas.0910834107. 20231430 PMC2851867

[psyp14752-bib-0052] Peirce, J. W. 2007. “PsychoPy—Psychophysics Software in Python.” Journal of Neuroscience Methods 162, no. 1/2: 8–13. 10.1016/j.jneumeth.2006.11.017. 17254636 PMC2018741

[psyp14752-bib-0053] Polich, J. 2007. “Updating P300: An Integrative Theory of P3a and P3b.” Clinical Neurophysiology 118, no. 10: 2128–2148. 10.1016/j.clinph.2007.04.019. 17573239 PMC2715154

[psyp14752-bib-0054] Quent, J. A. , A. Greve , and R. N. Henson . 2022. “Shape of U: The Nonmonotonic Relationship Between Object–Location Memory and Expectedness.” Psychological Science 33, no. 12: 2084–2097. 10.1177/09567976221109. 36221196

[psyp14752-bib-0055] Quent, J. A. , R. N. Henson , and A. Greve . 2021. “A Predictive Account of How Novelty Influences Declarative Memory.” Neurobiology of Learning and Memory 179: 107382. 10.1016/J.NLM.2021.107382. 33476747 PMC8024513

[psyp14752-bib-0056] Rangel‐Gomez, M. , and M. Meeter . 2013. “Electrophysiological Analysis of the Role of Novelty in the von Restorff Effect.” Brain and Behavior: A Cognitive Neuroscience Perspective 3, no. 2: 159–170. 10.1002/BRB3.112. PMC3607156 23531713

[psyp14752-bib-0057] Rugg, M. D. , and T. Curran . 2007. “Event‐Related Potentials and Recognition Memory.” Trends in Cognitive Sciences 11, no. 6: 251–257. 10.1016/j.tics.2007.04.004. 17481940

[psyp14752-bib-0058] Schapiro, A. C. , L. V. Kustner , and N. B. Turk‐Browne . 2012. “Shaping of Object Representations in the Human Medial Temporal Lobe Based on Temporal Regularities.” Current Biology 22, no. 17: 1622–1627. 10.1016/j.cub.2012.06.056. 22885059 PMC3443305

[psyp14752-bib-0059] Schomaker, J. , and M. Meeter . 2018. “Predicting the Unknown: Novelty Processing Depends on Expectations.” Brain Research 1694: 140–148. 10.1016/j.brainres.2018.05.008. 29758180

[psyp14752-bib-0060] Schönbrodt, F. D. , and E. J. Wagenmakers . 2018. “Bayes Factor Design Analysis: Planning for Compelling Evidence.” Psychonomic Bulletin & Review 25, no. 1: 128–142. 10.3758/s13423-017-1230-y. 28251595

[psyp14752-bib-0061] Sinclair, A. H. , G. M. Manalili , I. K. Brunec , R. Alison Adcock , and M. D. Barense . 2021. “Prediction Errors Disrupt Hippocampal Representations and Update Episodic Memories.” Proceedings of the National Academy of Sciences of the United States of America 118, no. 51: e2117625118. 10.1073/pnas.211762511. 34911768 PMC8713973

[psyp14752-bib-0062] Staresina, B. P. , J. Fell , A. T. A. Do Lam , N. Axmacher , and R. N. Henson . 2012. “Memory Signals Are Temporally Dissociated in and Across Human Hippocampus and Perirhinal Cortex.” Nature Neuroscience 15, no. 8: 1167–1173. 10.1038/nn.3154. 22751037 PMC3428860

[psyp14752-bib-0063] Staresina, B. P. , and M. Wimber . 2019. “A Neural Chronometry of Memory Recall.” Trends in Cognitive Sciences 23, no. 12: 1071–1085. 10.1016/J.TICS.2019.09.011. 31672429

[psyp14752-bib-0064] Stewardson, H. J. , and T. D. Sambrook . 2020. “Evidence for Parietal Reward Prediction Errors Using Great Grand Average Meta‐Analysis.” International Journal of Psychophysiology 152: 81–86. 10.1016/J.IJPSYCHO.2020.03.002. 32272127

[psyp14752-bib-0065] Theobald, M. , E. Galeano‐Keiner , and G. Brod . 2022. “Predicting vs. Guessing: The Role of Confidence for Pupillometric Markers of Curiosity and Surprise.” Cognition and Emotion 36, no. 4: 731–740. 10.1080/02699931.2022.2029733. 35077310

[psyp14752-bib-0066] Turan, G. , I. Ehrlich , Y. L. Shing , and S. Nolden . 2023. “From Generating to Violating Predictions: The Effects of Prediction Error on Episodic Memory.” 10.31234/osf.io/zm29a.

[psyp14752-bib-0067] Turk‐Browne, N. B. , M. G. Simon , and P. B. Sederberg . 2012. “Scene Representations in Parahippocampal Cortex Depend on Temporal Context.” Journal of Neuroscience 32, no. 21: 7202–7207. 10.1523/JNEUROSCI.0942-12.2012. 22623664 PMC3373994

[psyp14752-bib-0068] Van Kesteren, M. T. R. , D. J. Ruiter , G. Fernández , and R. N. Henson . 2012. “How Schema and Novelty Augment Memory Formation.” Trends in Neurosciences 35, no. 4: 211–219. 10.1016/j.tins.2012.02.001. 22398180

[psyp14752-bib-0069] Vidal‐Gran, C. , R. Sokoliuk , H. Bowman , and D. Cruse . 2020. “Strategic and Non‐strategic Semantic Expectations Hierarchically Modulate Neural Processing.” Eneuro 7, no. 5: ENEURO.0229‐20.2020. 10.1523/ENEURO.0229-20.2020. PMC7608692 33023884

[psyp14752-bib-0070] von Restorff, H. 1933. “Über Die Wirkung Yon Bereiehsbildungen Im Spurenfeld (The Effects of Field Formation in the Trace Field).” Psychologische Forschung 18: 299–342. 10.1007/BF02441202.

[psyp14752-bib-0071] Wahlheim, C. N. , M. L. Eisenberg , D. Stawarczyk , and J. M. Zacks . 2022. “Understanding Everyday Events: Predictive‐Looking Errors Drive Memory Updating.” Psychological Science 33, no. 5: 765–781. 10.1177/09567976211053596. 35439426 PMC9248286

[psyp14752-bib-0072] Wittmann, B. C. , N. Bunzeck , R. J. Dolan , and E. Düzel . 2007. “Anticipation of Novelty Recruits Reward System and Hippocampus While Promoting Recollection.” NeuroImage 38, no. 1: 194–202. 10.1016/J.NEUROIMAGE.2007.06.038. 17764976 PMC2706325

[psyp14752-bib-0073] Yacoby, A. , N. Reggev , and A. Maril . 2021. “Examining the Transition of Novel Information Toward Familiarity.” Neuropsychologia 161: 107993. 10.1016/j.neuropsychologia.2021.107993. 34411595

[psyp14752-bib-0074] Yonelinas, A. P. 2002. “The Nature of Recollection and Familiarity: A Review of 30 Years of Research.” Journal of Memory and Language 46, no. 3: 441–517. 10.1006/JMLA.2002.2864.

[psyp14752-bib-0075] Zacks, J. M. , N. K. Speer , K. M. Swallow , T. S. Braver , and J. R. Reynolds . 2007. “Event Perception: A Mind/Brain Perspective.” Psychological Bulletin 133, no. 2: 273–293. 10.1037/0033-2909.133.2.273. 17338600 PMC2852534

